# Host response profile of human brain proteome in toxoplasma encephalitis co-infected with HIV

**DOI:** 10.1186/1559-0275-11-39

**Published:** 2014-11-01

**Authors:** Apeksha Sahu, Satwant Kumar, Sreelakshmi K Sreenivasamurthy, Lakshmi Dhevi N Selvan, Anil K Madugundu, Soujanya D Yelamanchi, Vinuth N Puttamallesh, Gourav Dey, Abhijith K Anil, Anand Srinivasan, Kanchan K Mukherjee, Harsha Gowda, Parthasarathy Satishchandra, Anita Mahadevan, Akhilesh Pandey, Thottethodi Subrahmanya Keshava Prasad, Susarla Krishna Shankar

**Affiliations:** Institute of Bioinformatics, International Technology Park, Bangalore, 560066 India; Bioinformatics Centre, School of Life Sciences, Pondicherry University, Puducherry, 605014 India; Manipal University, Madhav Nagar, Manipal, 576104 India; Amrita School of Biotechnology, Amrita University, Kollam, 690525 India; School of Biotechnology, KIIT University, Bhubaneswar, 751024 India; Armed Forces Medical College, Pune, 411040 India; Department of Pharmacology, Postgraduate Institute of Medical Education & Research, Chandigarh, 160012 India; Department of Neurosurgery, Postgraduate Institute of Medical Education & Research, Chandigarh, 160012 India; Department of Neurology, National Institute of Mental Health and Neurosciences, Bangalore, 560029 India; Department of Neuropathology, National Institute of Mental Health and Neurosciences, Bangalore, 560029 India; Human Brain Tissue Repository, Neurobiology Research Centre, National Institute of Mental Health and Neurosciences, Bangalore, 560029 India; McKusick-Nathans Institute of Genetic Medicine, Johns Hopkins University School of Medicine, Baltimore, MD 21205 USA; Department of Biological Chemistry, Johns Hopkins University School of Medicine, Baltimore, MD 1205 USA; Department of Oncology, Johns Hopkins University School of Medicine, Baltimore, MD 21205 USA; The Sol Goldman Pancreatic Cancer Research Center, Department of Pathology, Johns Hopkins University School of Medicine, Baltimore, MD 21205 USA; NIMHANS-IOB Proteomics and Bioinformatics Laboratory, Neurobiology Research Centre, National Institute of Mental Health and Neurosciences, Bangalore, 560029 India

**Keywords:** iTRAQ labeling, Neuroinfections, Opportunistic infections, Chronic meningitis, Immunosuppression, LTQ-Orbitrap Velos

## Abstract

**Background:**

Toxoplasma encephalitis is caused by the opportunistic protozoan parasite *Toxoplasma gondii*. Primary infection with *T. gondii* in immunocompetent individuals remains largely asymptomatic. In contrast, in immunocompromised individuals, reactivation of the parasite results in severe complications and mortality. Molecular changes at the protein level in the host central nervous system and proteins associated with pathogenesis of toxoplasma encephalitis are largely unexplored. We used a global quantitative proteomic strategy to identify differentially regulated proteins and affected molecular networks in the human host during *T. gondii* infection with HIV co-infection.

**Results:**

We identified 3,496 proteins out of which 607 proteins were differentially expressed (≥1.5-fold) when frontal lobe of the brain from patients diagnosed with toxoplasma encephalitis was compared to control brain tissues. We validated differential expression of 3 proteins through immunohistochemistry, which was confirmed to be consistent with mass spectrometry analysis. Pathway analysis of differentially expressed proteins indicated deregulation of several pathways involved in antigen processing, immune response, neuronal growth, neurotransmitter transport and energy metabolism.

**Conclusions:**

Global quantitative proteomic approach adopted in this study generated a comparative proteome profile of brain tissues from toxoplasma encephalitis patients co-infected with HIV. Differentially expressed proteins include previously reported and several new proteins in the context of *T. gondii* and HIV infection, which can be further investigated. Molecular pathways identified to be associated with the disease should enhance our understanding of pathogenesis in toxoplasma encephalitis.

**Electronic supplementary material:**

The online version of this article (doi:10.1186/1559-0275-11-39) contains supplementary material, which is available to authorized users.

## Background

Toxoplasma encephalitis (TE) is a major complication of central nervous system caused by *Toxoplasma gondii,* an obligate intracellular protozoan. TE presents with highly varied neurological symptoms with both focal and diffuse neurological abnormalities. The risk of developing TE is high in immunocompromised individuals including patients with HIV infection, solid organ transplantation and those receiving immunosuppressive therapy, mostly due to recrudescence of latent infection [1–3].

Various diagnostic tests have been in practice for the diagnosis of toxoplasmosis in serum, CSF and brain tissues [3]. Conventionally, radiologic techniques such as CT scan and MRI scan are used for visualizing the lesions produced in TE [4]. Enzyme-linked immunosorbent assays to measure IgG and IgM levels against *T. gondii* in serum and urine are also routinely used in diagnostics [5]. Advanced diagnostic procedures including loop mediated isothermal amplification reactions and PCR-based assays are also used for the detection of *T. gondii* with a high degree of sensitivity [6].

The life cycle of *T. gondii* is divided into sexual and asexual phases. Sexual reproduction occurs in cats, which are the definitive hosts for this parasite. Gametocytes present in the intestinal epithelium of cats are fertilized and infectious oocysts are shed through feces. These oocysts sporulate in the environment and contaminate soil, water and food, through which infection is transmitted to warm blooded animals including humans. These warm blooded animals act as intermediate hosts, in which asexual reproduction occurs in the form of actively replicating tachyzoites. Host immune response induces conversion of tachyzoites to semi-dormant bradyzoites, which form tissue cysts. The life cycle of *T. gondii* is completed when tissue cysts are ingested again by cats [7].

Around one-third of the general population worldwide is considered to be infected with *T. gondii*, most of whom are asymptomatic [7]. The prevalence of infection depends on the climatic conditions and the age of the population. Studies in India have shown that ~25% of general population is seropositive for *T. gondii* IgG antibodies [3]. Transmission of *T. gondii* to humans may occur from an infected mother to the fetus or orally by consumption of either sporulated oocysts from contaminated soil, food or water or bradyzoites from undercooked or uncooked meat. Upon gaining entry into the human body, *T. gondii* gets converted into tachyzoite stage, crosses the gastrointestinal barrier to disseminate throughout the body including immune privileged sites such as brain, retina and fetus. Tachyzoites invade the astrocytes and microglial cells to breach the blood-brain barrier and form tissue cysts in the brain [8]. In immunocompromised individuals with <200 CD4+ cells per μl, the conversion of bradyzoites to tachyzoites leads to TE [1].

During acute infection and reactivation of latent *T. gondii* infection, the immune system challenges the parasite by producing interferon gamma (IFN-γ), tumor necrosis factor alpha (TNF-α) and inducible nitric oxide synthase (iNOS)*.* CD4+ T cells, CD8+ T cells and natural killer cells produce IFN-γ and TNF-α following *T. gondii* infection. IL-12, IL-6 and IL-4 also were shown to prevent formation of cysts and tachyzoite proliferation, probably through the maintenance of IFN-γ and TNF-α mediated resistance [9]. IFN- γ also induces the expression of vascular cell adhesion molecule-1 (VCAM-1), which enhances the recruitment of CD4+ T cells and CD8+ T cells to the site of infection [10]. Molecular mechanisms underlying the host-pathogen interactions have been studied using mouse models and cell lines with respect to TE. Though substantial information was provided by these studies, there still remain unanswered questions that emphasize the need for further studies at the molecular level in humans [11, 12]. Therefore, we carried out a global unbiased quantitative proteomic analysis of brain tissues from TE patients co-infected with HIV by iTRAQ labeling and high-resolution mass spectrometry using LTQ-Orbitrap Velos mass spectrometer. Differentially expressed proteins identified from TE brain tissues co-infected with HIV included several novel proteins along with a few proteins, which were reported earlier. We also validated three differentially expressed proteins using immunohistochemistry (IHC) and found that their expression was consistent with the mass spectrometry results.

## Results and discussion

**Figure 1:**
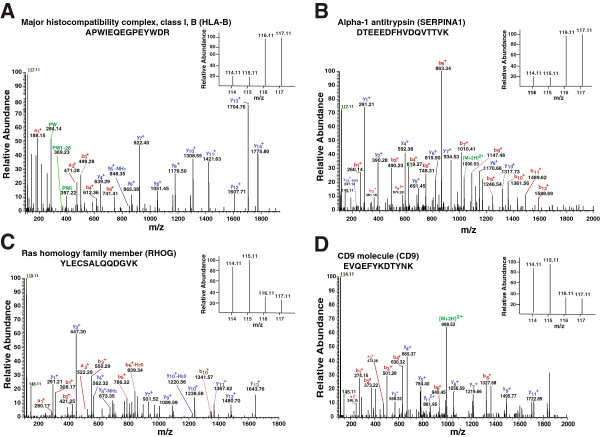
**Representative MS/MS spectra of peptides of differentially expressed proteins: A) Major histocompatibility complex, class I, B (HLA-B) protein is upregulated 4.4-fold in toxoplasma encephalitis brain tissues when compared to controls.** **B)** Alpha-1 antitrypsin (SERPINA1) protein is upregulated by 3.0-fold in toxoplasma encephalitis brain tissues when compared with control. **C)** Ras homolog family member G (RHOG) protein is downregulated by 2.5-fold in toxoplasma encephalitis brain tissues when compared with control. **D)** CD9 molecule (CD9) is downregulated by 2.0-fold in toxoplasma encephalitis brain tissues when compared with control.

We carried out a quantitative proteomic analysis of the frontal lobe brain tissues from TE patients co-infected with HIV and uninfected control subjects using iTRAQ labeling followed by SCX fractionation and high resolution Fourier transform mass spectrometry. The mass spectra were searched using SEQUEST search algorithm against Human RefSeq protein database version 52, which comprised of 33,987 protein sequences. In total, we identified 3,496 proteins of which 607 proteins were differentially expressed (≥1.5-fold). Among the differentially expressed proteins, 293 and 314 proteins were found to be overexpressed and downregulated, respectively, in brain tissues of TE patients in comparison to control brain tissues (Additional file 1: Table S1). A partial list of overexpressed and downregulated proteins is provided in Tables 1 and 2, respectively. Representative MS/MS spectra of selected proteins are provided in Figure 1. We have deposited the mass spectrometry data to Human Proteinpedia and PRIDE repositories [13, 14].

**Table 1:** **A partial list of upregulated regulated proteins in toxoplasma encephalitis**

	Gene symbol	Protein	Description	Toxoplasma encephalitis/Control (Fold change)
**1**	*HLAB*	Major histocompatibility complex, class I, B	It is a membrane protein and a member of HLA complex. It plays an important role in immune system by presenting antigens derived from endoplasmic reticulum.	4.4
**2**	*MPO*	Myeloperoxidase	It is a heme protein, expressed mainly in azurophilic granules of neutrophils. It produces hypohalous acid involved in killing of bacteria and pathogens.	3.8
**3**	*SERPINA1*	Alpha-1 antitrypsin	It is a member of serpin family. It acts as anti-trypsin inhibitor and its targets mainly include protein like elastase and chymotrysin	2.9
**4**	*CRP*	C-reactive protein	It belongs to pentraxin family. It is involved in a wide range of host related immune response reactions.	2.3
**5**	*ICAM-1*	Intercellular adhesion molecule 1	It is a glycoprotein, which acts as an intercellular adhesion molecule and is involved in pro-inflammatory pathways.	2.1
**6**	*GRIA3*	Glutamate receptor 3 isoform 1	It is a neurotransmitter receptor, which regulates neurophysiological process by acting as an excitatory neurotransmitter.	2.0
**7**	*CACNG2*	Voltage-dependent calcium channel gamma-2 subunit	It is an integral membrane protein, which mediates trafficking of AMPA receptor.	1.7
**8**	*SYT1*	Synaptotagmin-1	It is an integral membrane protein. It is involved in sensing of Ca2(+) during the process of vesicular trafficking and exocytosis.	1.8
**9**	*STXBP1*	Syntaxin-binding protein 1	It plays a significant role in neurotransmission and modulation of synaptic vesicle trafficking.	1.6
**10**	*SYN1*	Synapsin-1	It is a phosphoprotein involved in the inhibition of neurotransmission and regulation of synaptogenesis.	1.6

**Table 2:** **A partial list of downregulated regulated proteins in toxoplasma encephalitis**

	Gene symbol	Protein	Description	Control/Toxoplasma encephalitis (Fold change)
1	*ASPA*	Aspartoacylase	It catalyzes the hydrolysis of N-acetyl aspartate (NAA) to form aspartate and acetate. Accumulation of NAA due to deficiency of ASPA results in seizures and loss of previously acquired skills.	3.7
**2**	*PLP1*	Myelin proteolipid protein	It is a transmembrane proteolipid protein, which synthesizes and/or maintains multi lamellar structure of myelin.	3.4
**3**	*RHOG*	Rho-related GTP-binding protein, member G	It belongs to RHO family of small GTPase superfamily. It has a role in cytoskeleton arrangement and various Rho and Rac mediated signal transduction.	2.7
**4**	*CBR1*	Carbonyl reductase1	It is a monomeric enzyme, which metabolizes toxic environmental quinones and pharmacological substrates including anticancer doxorubicin.	2.6
**5**	*GPD1*	Glycerol-3-phosphate dehydrogenase	It has a major role in NADH oxidation, gluconeogenesis, triglyceride biosynthetic process and various other metabolic pathways.	2.4
**6**	VAT1	Synaptic vesicle membrane protein VAT-1	It belongs to quinone oxidoreductase subfamily under the family named zinc-containing alcohol dehydrogenase proteins. It inhibits mitochondrial fusion by acting along with mitofusin proteins.	2.3
**7**	*RAP1A*	Ras-related protein Rap-1A	It is a member of the Ras family of small GTPases, which is known to regulate cell-cell adhesion. Depletion of RAP1 is shown to increase endothelial permeability.	2.2
**8**	HSPA2	Heat shock-related 70 kDa protein 2	It belongs to heat shock protein 70 family. It has a role in synaptonemal complex disassembly.	2.2
**9**	*PRKAR1A*	cAMP-dependent protein kinase type I-alpha regulatory subunit	It belongs to cAMP-dependent kinase regulatory chain family having role in mesoderm formation, blood coagulation and various signal transduction pathways.	2.1
**10**	*CRYL1*	Lambda-crystallin homolog	It belongs to the family of oxidoreductase. It has a role in fatty acid metabolism.	1.7

### Categorization of differentially expressed proteins based on Gene Ontology annotation

**Figure 2:**
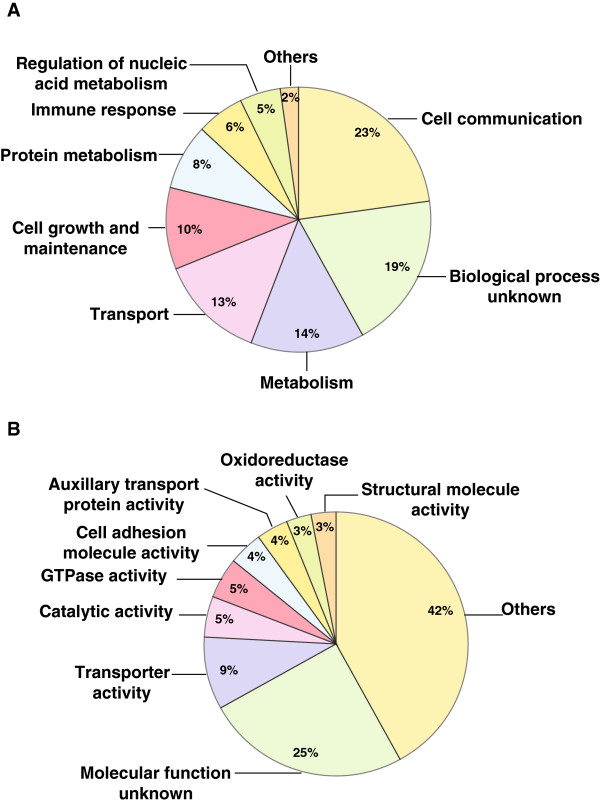
**Gene Ontology (GO) analysis of differentially regulated proteins:** **A) Biological processes-based analysis showed majority of the proteins involved in processes such as cell communication, growth and transport.** **B)** Molecular function-based analysis revealed a majority of the proteins with unknown functions. However, a significant number were seen with transporter and catalytic activities among others.

We categorized differentially expressed proteins based on their biological processes and molecular function using annotations in Human Protein Reference Database (HPRD; http://www.hprd.org) [15, 16], which follows Gene Ontology (GO) standards. Proteins involved in important biological processes such as cell communication (23%), metabolism/energy pathways (14%), transport (13%), cell growth/ maintenance (10%) and immune response (5%) were found to be altered in response to TE co-infected with HIV (Figure 2A). Classification of differentially expressed proteins based on their molecular functions enriched for proteins with catalytic activity (5%), transporter activity (9%), GTPase activity (5%), and cell adhesion activity (4%) (Figure 2B).

### Functional annotation of differentially expressed proteins in toxoplasma encephalitis

**Figure 3:**
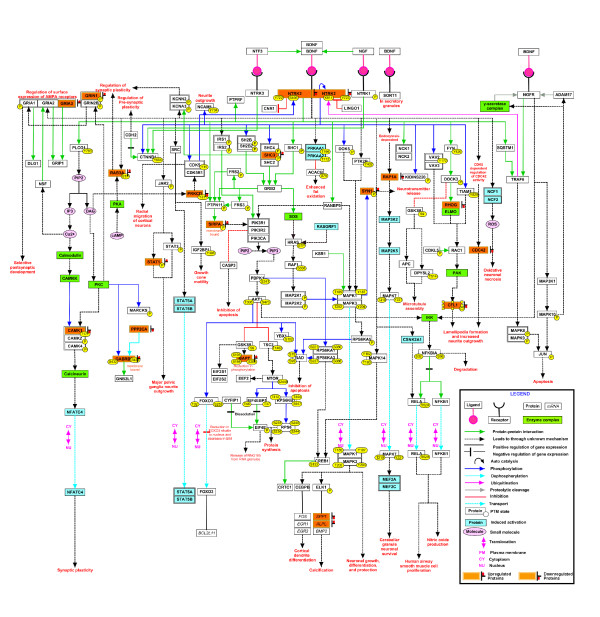
**Biological network analysis of differentially expressed proteins using GeneSpring identified deregulated BDNF pathway: Nineteen proteins involved in the BDNF pathway were differentially expressed including the downregulation of the BDNF receptor protein – NTRK2.** Proteins involved in BDNF mediated lamellipodia formation and neurite outgrowth were downregulated whereas proteins involved in BDNF mediated synaptic plasticity were overexpressed.

Pathway analysis of the differentially expressed proteins in TE co-infected with HIV brain tissue samples using the Pathway Architect module in GeneSpring (version 12.6) revealed the enrichment of several proteins involved in pathways such as neural transmission across chemical synapse, neurotransmitter release cycle, antigen processing and presentation along with complement and coagulation cascade pathway. In addition, several key modulators of the brain-derived neurotrophic factor (BDNF) signaling pathway were found to be differentially regulated, including aldo-keto reductase (AKR1C1), alkaline phosphatase (ALPL), secreted phosphoprotein (SPP1), heat shock protein (HSPA1A), chromogranin B (CHGB), ras homolog family member (RHOG), regulating synaptic membrane exocytosis (RIMS3), inositol polyphosphate-1-phosphatase (INPP1), integrin, alpha 2 (ITGA2), filamin C (FLNC) and polymerase I transcript release factor (PTRF) [17]. RHOG, which was found to be downregulated in TE compared to control tissues, is involved in mediating BDNF-induced neurite outgrowth [18]. It has been reported that HIV-1 promotes neuronal injury by reducing the length of neuronal processes through reduction in the mature BDNF levels in neurons [19]. Therefore, differential expression of several important molecules in BDNF signaling pathway may be the cause of cognitive aberration in HIV-associated TE (Figure 3).

### Biological significance of the differentially expressed human proteins

Differentially expressed proteins identified in the current study covers a diverse spectrum of functions including transmission across chemical synapses, antigen processing and presenting, neurotransmitter release cycle and interferon signaling. We identified proteins including guanine nucleotide binding protein (GNAO1), major histocompatibility complex class II protein (HLA-DRB1), heat shock 70 kDa protein 1A/1B, extracellular protein laminin (LAMA2), signal transducer and activation of transcription (STAT1), which have been previously reported to be associated with TE among the upregulated proteins. Calcium signaling by multiple G–protein effectors has been reported to be altered by inflammatory mediators, in inflammatory conditions in central nervous system (CNS) including infection, trauma, stroke, neurodegeneration and autoimmunity [20]. HLA-DRB1 is known to be a risk factor in HIV-1 patients for developing opportunistic infections, especially TE [21]. In addition, heat shock 70 kDa protein (HSPA1B) polymorphism has been shown to be associated with risk for schizophrenia, positive symptoms as well as clinical outcome [22, 23]. LAMA2 is an extracellular matrix protein, which regulates and promotes oligodendrocyte development, whereas STAT1 is actively modulated by *T. gondii* increasing the likelihood of its survival within the host [24–26].

### Host immune response proteins

Among the differentially expressed proteins, those involved in host immune response, such as complement proteins and major histocompatibility proteins were identified in this study. We detected several proteins reported in interferon-gamma signaling to be overexpressed, including guanylate binding protein (GBP1), major histocompatibility complex protein (HLA-B), intercellular adhesion molecule (ICAM1), ubiquitin-like modifier (ISG15) and STAT1. Microtubule-associated protein tau (MAPT), C-reactive protein (CRP) along with STAT1, involved in IL-2, IL-5 and IL-6 signaling pathways, were also found to be overexpressed in TE brain tissues. In patients with *T. gondii* and HIV infection, marked decrease in CD4+ T cells has been reported and often there is no significant increase in the antibody titer, as observed during aggravation of infection [27]. Furthermore, in T cells from HIV patients with toxoplasmosis, secretion of both interferon-gamma and IL-2 has been known to be altered [28]. Persistence of toxoplasma infection is contributed by aberrant production of these vital immune cytokines. Therefore, with the progressive depletion of the T cell dependent protective mechanisms in HIV-1, especially CD8+ T cells, probability of developing toxoplasma infection increases. However, in our study we observed overexpression of proteins associated with interferon-gamma and IL-2 signaling pathways, indicating restored immune response in the patients. Similar restoration of immune response has been reported by other studies in patients previously subjected to antiretroviral therapy [29, 30].

Other proteins involved in interferon signaling that were found to be overexpressed include beta-2 microglobulin (B2M), major histocompatibility complex protein (HLA-G), interferon-induced protein with tetratricopeptide repeats (IFIT1 and IFIT3) and myxovirus (influenza virus) resistance 1 interferon-inducible protein (MX1). B2M is important in diagnosis of HIV and its rise in HIV patients is considered as an indicator of HIV progression [31]. Increased HLA-G level is known to be associated with congenital transmission of *T. gondii* whereas, increased expression of IFIT1 has been reported in HIV-1 infected astrocytes [32, 33]. Overexpression of B2M, IFIT3, MX1 and STAT1 has been reported in the brains of neuro-AIDS patients, including those on combination antiretroviral therapy and also in non-human primate model of neuro-AIDS [34]. JAK/STAT pathway is known to be modulated by toxoplasma and involved in the pathogenesis of depression [35, 36]. It has also been shown that toxoplasmosis is associated with depression along with other psychiatric manifestations [37].

### Proteins in neurotransmitter release cycle and transmission across chemical synapses

In this study, several proteins playing an important role in neurotransmitter release cycle and transmission across chemical synapses were found to be differentially expressed. These include actin (ACTN2), calcium channel voltage-dependent gamma subunit 2 (CACNG2), DnaJ protein homolog (DNAJC5), gamma-aminobutyric acid receptors (GABBR1, GABBR2, GABRA1 and GABRG2), guanine nucleotide binding proteins (GNG13 and GNG4), glutamate receptors (GRIA3 and GRIN1), monoamine oxidase A (MAOA), member RAS oncogene family (RAB3A), solute carrier family proteins (SLC17A7, SLC32A1 and SLC6A1), syntaxins (STX1A and STXBP1), synapsins (SYN1, SYN2 and SYN3) and synaptotagmin (SYT1). Among these, CACNG2 has been reported to be associated with schizophrenia [38, 39]. DNAJC5 anchored to synaptic vesicles, along with exocrine, endocrine and neuroendocrine secretory granules, has been shown to have anti-neurodegenerative properties [40]. Interestingly, evidence of GABAergic dysfunction in lateral cerebella, due to reduced protein levels of both receptors GABBR1 and GABBR2, has been reported in subjects of schizophrenia, bipolar disorder and major depression [41]. Also, it has been shown that mutations in GABRA1 lead to epileptic encephalopathies and mutations in GABRG2 have been associated with generalized epilepsy [42–44]. Among overexpressed, glutamate receptors GRIA3 has a positive association in women patients with schizophrenia patients [45]. MAOA is known to catalyze the oxidative deamination of amines and has been implicated in antisocial behavior [46]. Alterations in solute carrier family proteins (SLC17A7, SLC32A1 and SLC6A1) implicating impairment of synaptic transmission may contribute in schizophrenia pathology [47]. Among syntaxin proteins (STX1A and STXBP1), STXBP1 was known to be a key molecule in ion channel regulation and synaptic exocytosis and has been reported widely in cases of migraine in different geographical settings [48]. Mutations in *STXBP1* gene are responsible for infantile epileptic encephalopathy-4 [49, 50]. *SYN1* gene has been reported as a susceptible gene for autism-spectrum disorder (ASD). Knockout mice study revealed that lack of Syn1, Syn2 and Syn3 cause cognitive impairment and activates strong epileptic phenotype in mice (Syn1 and/or Syn2) [51]. Synaptotagmin-1 (SYT1) triggers neurotransmitter release at synapse by binding to calcium (Ca2+) [52]. Therefore, differential expression of neurotransmitter release cycle and transmission across chemical synapses may predispose the patient suffering from TE infection to seizure activity and cognitive impairments. Moreover, a number of studies connect *T. gondii* with higher incidence of schizophrenia [53, 54].

### Validation of differentially expressed proteins by immunohistochemistry

**Figure 4:**
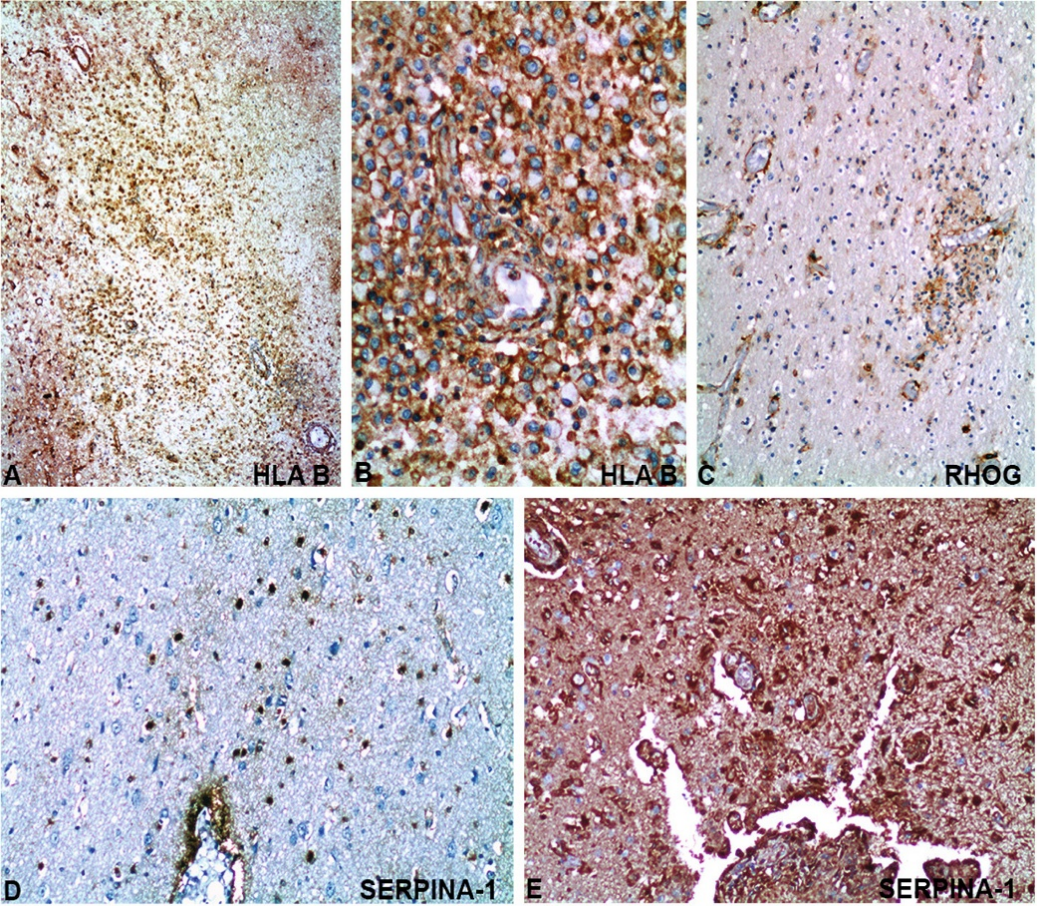
**Immunohistochemistry-based validation of differentially expressed proteins in toxoplasma encephalitis co-infected with HIV: A) Histiocytic elements in the toxoplasma lesion strongly expressed HLA-B, while the pale zones represent the necrotic acellular zones, B) Higher magnification highlights strong expression of HLA-B in the perivascular histiocytes, C) The expression of RHOG is marginally downregulated in the histiocytes and newly formed capillary endothelia, D) Control brain has low expression of alpha-1 antitrypsin (SERPINA1) in glial cells and a necrotic vessel wall, E) In the case of toxoplasma encephalitis the glial cells, the micro vessels and histiocytes expressed alpha-1 antitrypsin strongly unlike the control.**

Differentially expressed proteins identified using high resolution mass spectrometry from frontal lobe tissues of TE were validated using IHC. For this, we selected three candidates - HLA-B, SERPINA1 and RHOG - for IHC validation based on the molecular function of the proteins and its significance to the disease manifestation (Figure 4).

### Major histocompatibility complex, class I (HLA-B)

HLA-B protein known to be present on surface of nucleated cells is involved in antigen presentation to cytotoxic T cells. Earlier studies have shown that infection with *T. gondii* elicits HLA-B expression in dendritic cells, astrocytes and macrophages [55]. *Toxoplasma* antigens induce significant HLA-B expression in murine TE brain tissue, which may play role in *Toxoplasma* antigen presentation evoking active immune response [56]. We found upregulation of HLA-B in HIV-associated TE brain tissues. Immunohistochemical staining showed strong HLA-B expression in histiocytic elements of toxoplasma lesion, while the pale zones represent the necrotic acellular zones. Strong expression of HLA-B was observed in the perivascular histiocytes at higher magnification.

### Alpha-1 antitrypsin (SERPINA1)

Alpha-1 antitrypsin (SERPINA1) is a member of the serpin family. Polymorphisms in the *SERPINA1* gene have been reported to be associated with fibromyalgia and mood disorders [57]. Hypersialylated SERPINA1 has been reported to be a protein of significant diagnostic value for early detection of Parkinson’s dementia [58]. It has also been associated with other neurological disorders like Alzheimer’s disease and schizophrenia [59, 60]. It is interesting to note that serine protease inhibitor neuroserpin regulates the growth and maturation of hippocampal neurons [61] dysfunction of hippocampus is one of the critical factors in the pathogenesis of schizophrenia [62]. Apart from this alpha-1 antitrypsin blocks the processing of group antigens (Gag) and envelop proteins (Env), which results in suppression of HIV-1 morphogenesis [63]. Immunohistochemical labeling showed strongly expressed alpha-1 antitrypsin in the glial cells, the micro vessels and histiocytes of toxoplasma encephalitis brain tissues unlike in the control brain tissues, which showed low expression in glial cells and the necrotic vessel wall.

### Ras homolog family member G (RHOG)

RHOG is mainly involved in actin cytoskeleton rearrangement, cell migration and protection of cell from anoikis [64, 65]. Recent studies unraveled the role of RHOG in pathogenesis of bacterial infections [66]. It has been detected in rat hippocampus, cerebellum and specific expression was observed in oligodendrocytes and neuronal cells of brain [67, 68]. As reported by multiple studies RHOG has significant role in various neurological processes including neurite extension, spine morphogenesis, neuronal progenitor cell proliferation and axonal outgrowth [69, 70]. We observed down-regulation of RHOG in TE brain tissues co-infected with HIV. Immunohistochemical labeling showed the marginal downregulation of RHOG expression in the histiocytes and newly formed capillary endothelia.

## Conclusions

TE is a principal opportunistic infection of the CNS in individuals infected with HIV. The molecular profile associated with pathological progression of TE has not been explored using a global proteomics platform. Utilizing an iTRAQ-based quantitative proteomic approach, we identified a subset of proteins, which are differentially expressed in TE co-infected with HIV brain tissues. Our data provides insights into molecular profiles of TE, which can be investigated through several hypotheses. We found differential regulation of key proteins involved in host-immune response and neurotransmitter release cycle, neuronal growth, energy metabolism and calcium transport. Increase in the neurotransmission across synapses may predispose the patients to seizure activity and cognitive impairment in the clinical evolution of TE. We also identified overexpression of proteins, which may have a role in the development of schizophrenia among TE patients. Upregulation of caveolar proteins and intercellular adhesion molecules indicate their possible role along with gp41 protein from HIV in mediating the entry of the pathogens by breaching blood-brain barrier.

## Methods

### Sample collection

The study was approved by scientific ethics committee of National Institute of Mental Health and Neuro Sciences (NIMHANS), Bangalore, India. Brain tissue samples from the lesion site within the frontal lobe from five confirmed cases of cerebral toxoplasmosis associated with HIV-1 infection were collected during autopsy and divided into symmetric halves. One half was processed for histopathological studies including immunohistochemistry for toxoplasma and macrophage response (CD 68), while the other half was used for proteomic study. All tissues included in the study revealed features of toxoplasma encephalitis in the cortical ribbon and subcortical zone with areas of necrosis, inflammation and variable CD 68 positive histocytic response along with the presence of toxoplasma organisms (both bradyzoites and tachyzoites). All cases were positive for HIV-1 on serological testing and confirmed to be HIV-1 clade C by clade specific PCR developed at Jawaharlal Nehru Centre for Advanced Scientific Research in Bangalore and validated. Five uninfected control brain tissues from the similar age and sex group were collected from victims of road traffic accidents within 10-19 h post mortem during autopsy (Additional file 2: Tables S2 and S3). Fifteen toxoplasma encephalitis frontal cortex brain tissue samples including five samples taken for proteomics study from age group 1 to 54 years and one uninfected control sample were included for immunohistochemistry validation. The autopsy was conducted within 3-19 hours and bodies were kept at 4°C after death. The control tissues were confirmed to be negative for HIV-1 and toxoplasma infection. The samples were obtained from the archives of Human Brain Tissue Repository, Department of Neuropathology, NIMHANS, Bangalore.

### Protein extraction and normalization

Around 100 mg of frontal lobe brain tissue from each individual were homogenized by crushing in liquid nitrogen with the aid of a mortar and pestle. Once the tissues were ground to fine powder, around 1 ml of 0.5% SDS was added and homogenized. Tissue lysate was transferred to sterile microcentrifuge tubes and centrifuged at 14,000 × g for 30 min at 4°C. Clear supernatant was collected in separate microcentrifuge tubes. Total protein estimation was carried out using BCA protein assay (Pierce, Thermo Scientific). Normalization of protein amounts was confirmed using 10% SDS-PAGE. Equivalent amounts of protein from each of the five TE with HIV-1 samples were pooled and similar processing was done for control samples.

### iTRAQ labeling and strong cation exchange chromatography

Pooled protein samples from each condition were reduced, alkylated and digested with trypsin. iTRAQ labeling (Applied Biosystems, cat# - 4352135) of peptides was carried out according to manufacturer’s protocol as described earlier [71]. Briefly, 200 μg of protein from each condition was treated with 4 μl of reducing agent (tris (2-carboxyethyl) phosphine) at 60°C for 1 hour and alkylation of cysteine residues was carried out by incubating the samples with 2 μl of methyl methanosulfonate for 10 minutes at room temperature. Alkylated proteins were then digested using sequencing grade trypsin (Promega, cat# - V511A) with an enzyme to substrate ratio of 1:20 (w/w) at 37°C for 16 hours. Peptides from each condition were split into two equal halves and iTRAQ labeling was carried out in replicates. Peptides from control samples was labeled using iTRAQ reagent yielding reporter ions 114 and 115, peptides from TE with HIV-1 patient samples was labeled with iTRAQ reagents yielding reporter ions 116 and 117. Labeling was carried out by incubating the peptide-label mixture at room temperature for one hour, the reaction was quenched by addition of 150 μl milliQ water followed by incubation at room temperature for one hour.

iTRAQ labeled peptides from control and TE with HIV-1 were pooled, vacuum dried and reconstituted in 5 mM potassium phosphate buffer, 25% acetonitrile (pH 2.8) (solvent A). Strong cation exchange chromatography was carried out as described earlier [72]. The peptides were fractionated on polysulfoethyl A column (PolyLC, Columbia, MD) (100 × 2.1 mm, 5 μm particles with 300 Å pores) Agilent 1200 infinity series HPLC system for a period of 50 minutes using a gradient of increasing salt concentration of up to 350 mM KCl in solvent A. The fractions collected were pooled based on SCX profile into 21 fractions, dried and reconstituted in 40 μl of 0.1% formic acid. Samples were desalted using C_18_ (3 M Empore high-performance extraction disks) stage tip dried and stored in -20°C till mass spectrometric analysis.

### LC-MS/MS analysis

LC-MS/MS analysis was carried out on LTQ Orbitrap Velos ETD – mass spectrometer (Thermo Scientific, Bremen, Germany) interfaced with Proxeon nanoLCII (Thermo Scientific, Bremen, Germany). Peptides were analyzed on a Reversed Phase Liquid Chromatography (RPLC). The RPLC system equipped with a pre-column (2 cm, 5 μ – 100 Å) and an analytical column (12 cm, 3 μ – 100 Å) made with magic AQ C18 material (Michrom Bioresources, Inc, Auburn, CA) packed in-house. Further, the peptides are sprayed using 10 μ nano electro spray emitter tip (New Objective, Woburn, MA) fixed to an NSI source. The peptides were loaded on the pre column using solvent A (0.1% formic acid, 5% acetonitrile and resolved on the analytical column using a linear gradient of 8-30% solvent B (95% acetonitrile, 0.1% formic acid) for 75 minutes at a constant flow rate of 0.35 μl/min. The spray voltage and heated capillary temperature were set to 2.2 kV and 250°C, respectively. The MS spectra were acquired in a data-dependent manner in the m/z range of 350 to 1800. From each MS survey scan, 15 most intense precursor ions were selected for fragmentation. MS and MS/MS scans were acquired in an Orbitrap mass analyzer and the peptides were fragmented by higher energy collision dissociation with normalized collision energy of 41%. MS scans were acquired at a resolution of 60,000 at 400 m/z, while MS/MS scans were acquired at a resolution of 15,000. The automatic gain control (AGC) for full FT MS was set to 1 million ions and for FT MS/MS was set to 0.1 million ions with a maximum time of accumulation of 200 ms and 500 ms, respectively.

### Data analysis

The mass spectrometry data obtained was searched against the human RefSeq 52 proteins using Proteome Discoverer, version 1.3.0.339 (Thermo Scientific, Bremen, Germany) workflow. The workflow consisted of spectrum selector, reporter ion quantifier and SEQUEST nodes along with peptide validator node. Search parameters included trypsin as the enzyme with 1 missed cleavage; oxidation of methionine was set as dynamic modification, while methylthio modification of cysteine and iTRAQ modification at N-terminus of the peptide and lysine were set as static modifications. Precursor and fragment mass tolerance were set to 20 ppm and 0.1 Da, respectively with a signal to noise ratio of 1.5 for a precursor mass range of 350-10,000 Da. The raw data obtained was searched against decoy database to calculate 1% false discovery rate cut-off score. Spectra that matched to the contaminants and those that did not pass the 1% FDR threshold were not considered for analysis.

### Bioinformatics analysis

Bioinformatics analysis was carried out to categorize proteins based on biological processes, cellular component and molecular function using annotations in Human Protein Reference Database (HPRD) [15, 16], which is in compliance with gene ontology (GO) standards. The differentially expressed proteins having ≥1.5 fold change were taken as input and biological network was generated using Pathway Architect module of GeneSpring version 12.6 (Agilent Biosystems, Santa Clara, CA). We also integrated the interaction data from NetPath (http://www.netpath.org) and HPRD (http://www.hprd.org) in the analysis using GeneSpring software as described earlier [17, 72, 73].

### Immunohistochemical labeling

Validation of differentially expressed proteins in TE co-infected with HIV-1 was carried out by immunohistochemical staining for 15 toxoplasma encephalitis cases and 6 controls using commercially available antibodies. Formalin fixed and paraffin embedded autopsy tissues were collected and cut into 5 μm thick sections on glass slides. These slides were subjected for deparaffinization and rehydration. Endogenous peroxidase activity was quenched by 3% H_2_O_2_ for 20 min at room temperature. Heat induced antigen retrieval was carried out using pressure cooker by placing the tissue sections in citrate buffer (pH 6.0). The tissue sections were incubated with 3% skimmed milk in PBS, pH 7.4 at room temperature followed by incubation with primary antibodies at following dilutions – anti-major histocompatibility complex, class I, B (dilution 1:200, cat # sc-55582, Santa Cruz), anti-alpha-1-antitrypsin (dilution 1:2000, cat # HPA00927-100 μl, Sigma-Aldrich), anti-rho-related GTP-binding protein RhoG (dilution 1:50, cat # HPA039871-100UL, Sigma-Aldrich) for two hours at room temperature. In parallel with test slides, appropriate negative and positive controls were also subjected for incubation at room temperature, followed by incubation with pre-diluted secondary antibody conjugated with poly HRP (catalog # QD630-XAKE) from BioGenex. The reaction was visualized with chromogen substrate DAB/H2O2 as per manufacturer’s instructions. The sections were counterstained with hematoxylin. The immunolabeled sections were examined and staining pattern, intensity, subcellular localization were visually scored by two expert neuropathologists - AM and SKS (NIMHANS).

### Data availability

We submitted the mass spectrometry data to Human Proteinpedia to make it available to the public [13]. The study design, experimental details, list of proteins and peptides identified in the study can be accessed using the following URL: http://www.humanproteinpedia.org/data_display?exp_id=00707. The mass spectrometry proteomics data have also been deposited to the ProteomeXchange Consortium [14] via the PRIDE partner repository with the dataset identifier PXD000261.

## Electronic supplementary material

Additional file 1: Table S1: A complete list of proteins along with peptides identified from toxoplasma encephalitis and control brain tissues. (XLSX 3 MB)

Additional file 2: Table S2: Details of samples used for quantitative proteomic analysis of toxoplasma encephalitis. **Table S3.** Details of samples used for immunohistochemistry-based validation of differentially expressed molecules from quantitative proteomic analysis of toxoplasma encephalitis co-infected with HIV. (XLSX 13 KB)

## Abbreviations

CT: Computed tomography
CSF: Cerebrospinal fluid
CNS: Central nervous system
FDR: False discovery rate
iTRAQ: Isobaric tag for relative and absolute quantitation
IHC: Immunohistochemistry
HPLC: High performance liquid chromatography
HIV: Human immunodeficiency virus
MRI: Magnetic resonance imaging
PCR: Polymerase chain reaction
SCX: Strong cation exchange
TE: Toxoplasma encephalitis.
